# Cortical GABA Levels Are Reduced in Post-Acute COVID-19 Syndrome

**DOI:** 10.3390/brainsci13121666

**Published:** 2023-12-01

**Authors:** Ksenija Marinkovic, David R. White, Austin Alderson Myers, Katie S. Parker, Donatello Arienzo, Graeme F. Mason

**Affiliations:** 1 Spatio-Temporal Brain Imaging Lab, Department of Psychology, San Diego State University, San Diego, CA 92182, USAabaldersonmyers@health.ucsd.edu (A.A.M.); darienzo@health.ucsd.edu (D.A.); 2 Department of Radiology, University of California, San Diego, CA 92093, USA; 3 Department of Psychiatry, University of California, San Diego, CA 92093, USA; 4 Department of Radiology and Biomedical Imaging, Psychiatry, and Biomedical Engineering, Yale University, New Haven, CT 06520, USA; graeme.mason@yale.edu

**Keywords:** magnetic resonance spectroscopy, ^1^H-MRS, GABA, NAA, long COVID, depression, insomnia, anxiety, cognitive deficits, excitation/inhibition balance

## Abstract

After recovering from the acute COVID-19 illness, a substantial proportion of people continue experiencing post-acute sequelae of COVID-19 (PASC), also termed “long COVID”. Their quality of life is adversely impacted by persistent cognitive dysfunction and affective distress, but the underlying neural mechanisms are poorly understood. The present study recruited a group of mostly young, previously healthy adults (24.4 ± 5.2 years of age) who experienced PASC for almost 6 months following a mild acute COVID-19 illness. Confirming prior evidence, they reported noticeable memory and attention deficits, brain fog, depression/anxiety, fatigue, and other symptoms potentially suggestive of excitation/inhibition imbalance. Proton magnetic resonance spectroscopy (^1^H-MRS) was used to examine the neurochemical aspects of cell signaling with an emphasis on GABA levels in the occipital cortex. The PASC participants were compared to a control (CNT) group matched in demographics, intelligence, and an array of other variables. Controlling for tissue composition, biological sex, and alcohol intake, the PASC group had lower GABA+/water than CNT, which correlated with depression and poor sleep quality. The mediation analysis revealed that the impact of PASC on depression was partly mediated by lower GABA+/water, indicative of cortical hyperexcitability as an underlying mechanism. In addition, N-acetylaspartate (NAA) tended to be lower in the PASC group, possibly suggesting compromised neuronal integrity. Persistent neuroinflammation may contribute to the pathogenesis of PASC-related neurocognitive dysfunction.

## 1. Introduction

The COVID-19 pandemic has imposed devastating hardship worldwide, including the tragic loss of life, unprecedented economic/financial burden, and a mental health crisis [1,2]. While the acute symptoms of COVID-19 are quite well-documented, for a notable subset of people, health problems can linger for a long time after recovering from the acute illness. A set of symptoms persisting at least four weeks [3,4,5] or two months or longer [6] after the initial infection is colloquially known as “long COVID”. However, the symptoms, termed post-acute sequelae of COVID-19 infection (PASC), often last much longer [6,7,8,9], even in people with a mild acute presentation [10,11,12], with some sequelae persisting for two years after infection [13]. Neurocognitive symptoms are disproportionally represented and include cognitive dysfunction (i.e., “brain fog”, memory and attention problems), insomnia, depression, anxiety, and chronic fatigue, among others [3,4,5,10,12,14,15,16,17,18]. Given the significant impact of PASC on the neurofunctional status and quality of life of many people over a prolonged period of time, treating this multidimensional condition has imposed a very heavy burden on our health system and other related services [15,19,20].

Belying its initial classification as a severe acute respiratory syndrome coronavirus (SARS-CoV-2), it has become clear that the virus affects many organ systems and that it exerts direct as well as secondary effects on the brain [19]. The virus has a strong affinity for angiotensin-conversion enzyme 2 (ACE2) receptor, which is expressed throughout the organism, including neurons and glial cells in multiple brain structures [20,21]. Furthermore, COVID-19 impacts the brain indirectly through a number of pathways [19,22], including immune dysregulation mediated by excessive cytokine production and inflammatory processes, which seem to play an important role in PASC pathogenesis. Indeed, neuroinflammation has been one of the proposed mechanisms contributing to neural hyperexcitability [23,24,25,26], which is reflected in neurocognitive PASC symptoms including depression, anxiety, insomnia, cognitive dysfunction, and others [27,28,29,30,31,32,33,34,35].

However, the neural underpinnings of these PASC symptoms are poorly understood. At the core of the problem is an inadequate understanding of the basic mechanisms underlying neural hyperexcitability and the scarcity of objective measures, as imaging data are lacking. As a dynamic, interactive system [36], the brain relies on an optimal excitation/inhibition (E/I) balance, which underlies local neural activity and long-range communication in healthy cognition [37,38,39,40,41]. Conversely, E/I imbalance is thought to lead to neuropsychiatric disorders [41,42,43,44,45,46]. E/I balance is governed by cell signaling, which has both neuroelectric and neurochemical aspects and can be examined with complementary measures. For instance, EEG signals reflect postsynaptic currents directly and in real time [47]. In contrast, proton magnetic resonance spectroscopy (^1^H-MRS) can provide insights into the neurochemical environment by quantifying the concentration of brain metabolites in vivo [48,49,50,51,52,53].

As the principal inhibitory neurotransmitter, GABA plays an essential role in modulating neurotransmission in the brain by ensuring a stable neural network organization and the optimal E/I balance important for the regulation of behavioral and cognitive functions [38,54,55,56,57,58,59]. At the same time, E/I imbalance associated with GABA dysfunction is thought to underlie a number of neuropsychiatric disorders [41,44,46,60,61]. Reliable detection of GABA in the neural tissue is impeded by its low concentration and its spectral overlap with the resonances of metabolites with stronger signals [52]. However, GABA levels can be measured with dedicated spectral editing methods such as Mescher–Garwood Point Resolved Spectroscopy (MEGA-PRESS) [62]. Based on J-coupling, this method quantifies GABA+ and acknowledges the contributions of co-edited macromolecules with resonances that overlap with the GABA signal [53,63]. As demonstrated in our recent study [64], GABA+ is commonly expressed relative to a water reference (GABA+/w) to avoid issues with creatine instability [65].

^1^H-MRS evidence on PASC is limited to two studies that compared PASC and control groups. The only available study that measured GABA+ used a Hadamard Encoding and Reconstruction of Mega-Edited Spectroscopy (HERMES) editing method to measure GABA+ levels in the frontal lobe as a function of PASC symptoms persisting for ~7 months on average [66]. GABA+ levels did not differ between the PASC and control groups, which comprised middle-aged individuals reporting a number of comorbid conditions prior to COVID-19. Clearly, additional evidence is needed, especially given that lower GABA+ levels have been reported in depression [67,68,69,70,71], which is highly prevalent in PASC, and given that neuroinflammation downregulates GABAergic function [72]. Furthermore, it has been shown that GABA agonists exert anti-inflammatory influence by reducing the severity of COVID-19 in a mouse model [73]. Increased neural hyperexcitability is a possible interpretation of another ^1^H-MRS study that reported higher Glx levels in people with PASC [74], with Glx representing pooled resonances arising from glutamate (Glu) and glutamine (Gln) [75].

Another metabolite with relevance to PASC is N-acetylaspartate (NAA) which, combined with small contributions from N-acetyl-aspartyl-glutamate (NAAG), shows the largest ^1^H spectrum peak with a resonance at 2.02 ppm [52,76,77]. NAA is synthesized in the neuronal mitochondria and is involved in metabolic processes supporting cell signaling [78]. Based on its sensitivity to neuronal dysfunction, NAA has been used as a marker of neuronal integrity, viability, density, and metabolic homeostasis [78,79]. Indeed, reduced NAA has been reliably reported for traumatic brain injury [80], depression [70,81,82], neuroinflammation [83], and a range of brain-based disorders [84]. 

Given the exceedingly scant imaging evidence, the present study used ^1^H-MRS to examine the impact of PASC on GABA levels and the neurochemical profile in general and behavioral self-reports and assessments in previously healthy adults who reported experiencing PASC for almost 6 months on average.

## 2. Materials and Methods

### 2.1. Participants

Eighteen mostly young adults (24.4 ± 5.2 years of age, 11 women) with PASC symptoms were recruited from the local community. They reported experiencing PASC (i.e., long COVID) symptoms for two months or longer, which conforms to the World Health Organization definition [6] and exceeds the four-week minimum duration proposed by the Centers for Disease Control and Prevention [3] and the National Institutes of Health [4]. All participants were in good health prior to contracting a mild acute COVID-19 illness confirmed by a positive test 25.39 ± 17.22 weeks before the scan. None of the participants required hospitalization during acute illness, nor as they continued experiencing PASC symptoms. All participants were right-handed and reported no history of concussions, seizures, neurological or psychiatric disorders, hearing or vision problems, regular tobacco or marijuana use or use within the past month, and no regular use of illicit drugs or use within the previous two months. Two PASC participants reported using Prozac (fluoxetine, a selective serotonin reuptake inhibitor) and Vyvanse (lisdexamfetamine), respectively. They omitted their medication at least 24 h before the scan. As described in greater detail below, these medications did not appear to affect the results even though they upregulate GABA function, against the hypothesized GABA reduction [70,85,86].

**Figure 1:**
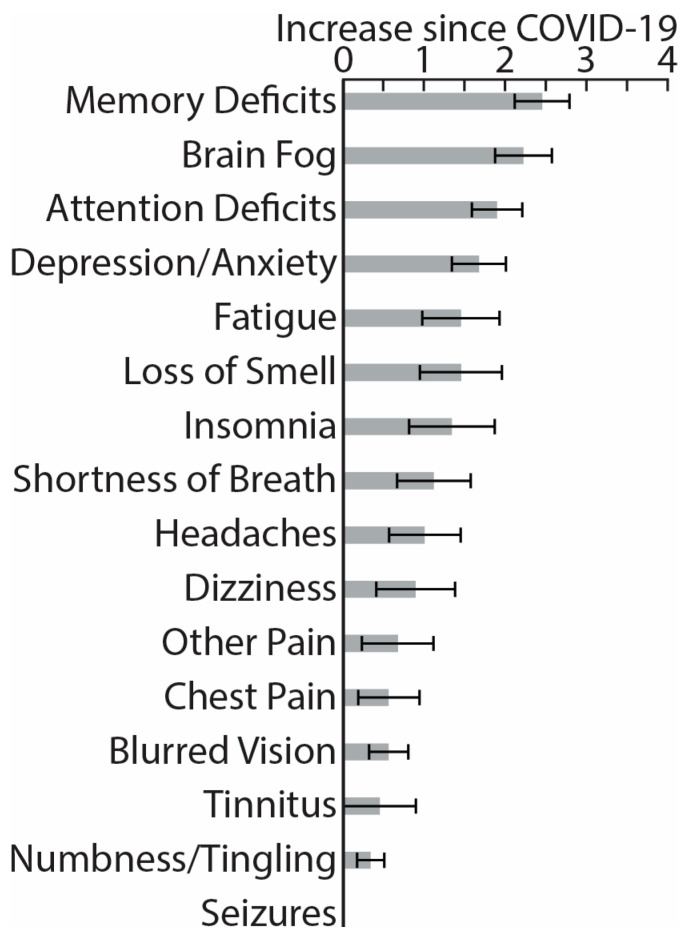
Histograms depict average increase in symptom severity on a scale from 0 (not at all) to 4 (very much) since recovering from the acute COVID-19 illness (means ± standard errors).

**Figure 2:**
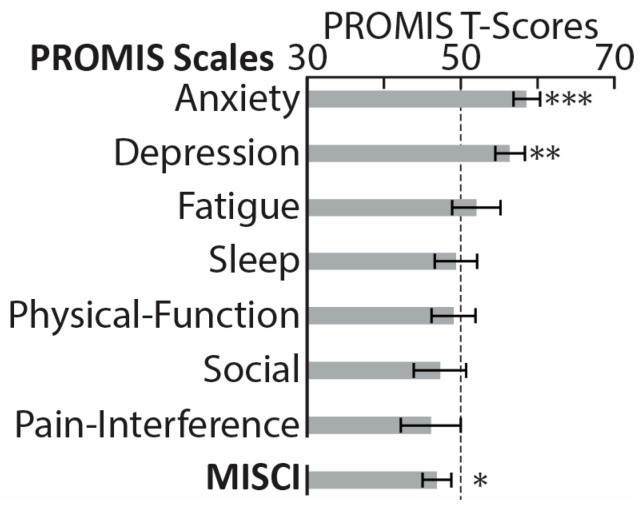
PROMIS (Patient-Reported Outcomes Measurement Information System) [91] comprises a set of short-form scales used to assess seven health domains in PASC (post-acute sequelae of COVID-19) participants. The scores were standardized with a T-score metric based on a normative sample with a mean of 50 (marked with a dotted line) and standard deviation of 10 [96]. PASC participants reported higher anxiety and depression on the two PROMIS scales. The PASC group also reported greater subjective cognitive impairment, as reflected in lower scores on MISCI (Multidimensional Inventory of Subjective Cognitive Impairment) [93], here shown after conversion to PROMIS-compatible T-scores. * *p* < 0.05, ** *p* < 0.01, *** *p* < 0.001.

While experiencing the PASC symptoms, participants reported negligible functional limitations in their daily duties and activities, 0.83 ± 1.34, on the Post-COVID-19 Functional Status Scale ranging from 0 (no functional limitations) to 4 (severe functional limitation) [87]. Similarly, on a Likert scale from 0 (not at all) to 4 (very much), they reported that PASC “somewhat” impacted their daily life, 1.7 ± 1.2. Figure 1 illustrates PASC symptoms in terms of their perceived increase since recovering from COVID-19, ranging from 0 (not at all) to 4 (very much). The most noticeable deterioration was reflected in memory deficits, brain fog, attention deficits, depression/anxiety, fatigue, etc., which is broadly consistent with previously reported evidence [9,14,34,88,89,90]. Based on Patient-Reported Outcomes Measurement Information System (PROMIS) scales [91], our PASC cohort reported greater depression and anxiety compared to the general population norms (Figure 2), confirming extensive similar evidence [12,16,33,88,89,90,92]. Furthermore, they had lower scores on the Multidimensional Inventory of Subjective Cognitive Impairment (MISCI) [93] than the population norms, which is indicative of greater cognitive deficits. This finding is aligned with numerous reports of cognitive dysfunction associated with PASC [89,90,94,95]. Overall, this pattern of deficits is consistent with lingering cognitive complaints colloquially termed “brain fog”, referring to long-term neurologic sequelae known as neuro-PASC [32]. 

The study’s procedures were approved by the San Diego State University Institutional Review Board. All participants provided written informed consent to participate in this protocol and received monetary compensation for their involvement.

The control group (CNT) comprised twenty participants (23.3 ± 3.7 years of age, 14 women) who had no medical concerns at the time of the study and reported no history of concussions, seizures, neuropsychiatric disorders, or hearing or vision problems. They were recruited from the same community as the PASC group, but they did not experience COVID-19. Half of the CNT sample was scanned before the lockdown. The CNT and PASC groups were matched on demographic variables (Table 1) and did not differ on intelligence, impulsivity, sensation seeking, stress, depression, or generalized anxiety. However, the PASC group reported worse sleep quality, which aligns with previous reports [34,97,98,99]. They also reported higher weekly drinking levels, which is consistent with increased prevalence of daily drinking in this age group during the pandemic and its common use as a coping mechanism [100,101].

### 2.2. Procedure

PASC participants were recruited from the local community through approved postings and ads. Upon expressing their interest in the study, prospective participants completed a screening questionnaire and were interviewed by a staff member. They were queried about the details of their acute illness, including its onset, symptom characteristics, duration, possible hospitalization, and positive confirmation with COVID-19 tests; the quality and duration of their PASC symptoms; the severity of PASC-related functional limitations in terms of daily activities [87]; the overall impact of PASC on their quality of life; and other comorbidities. Prospective participants were excluded if they were hospitalized during the acute COVID-19 illness to avoid confounding neurocognitive deficits with hospitalization-induced sequelae. They were excluded if they reported a history of head injury leading to loss of consciousness longer than 5 min, a neurological or psychiatric disorder, chronic health conditions preceding COVID-19 that were still ongoing, using illegal substances regularly or having used them in the previous two months, or smoking tobacco or marijuana regularly or in the previous month. Only the otherwise-eligible participants who reported PASC symptoms persisting for two months or longer were enrolled in the study.

Eligible participants completed a battery of assessments hosted by Qualtrics software, v 06/23 [102]. They were asked to rate the change in severity of the following symptoms since recovering from the acute illness (Figure 1): memory deficits, brain fog, attention deficits, depression/anxiety, fatigue, loss of smell, insomnia, shortness of breath, headaches, dizziness, chest pain, other pain, blurred vision, tinnitus, numbness/tingling, and seizures, modeled after previous studies [9,14,15,16,34,88,89,90]. They completed the PROMIS (Patient-Reported Outcomes Measurement Information System) [91], which measures self-reported pain interference, fatigue, physical functioning, depressive symptoms, anxiety, sleep disturbances, and ability to take part in social activities (Figure 2). The normalized T-scores were compared to the standard T-scores obtained from a normative sample (U.S. general population) with a mean of 50 and standard deviation of 10 [96]. Participants rated their cognitive dysfunction with MISCI (Multidimensional Inventory of Subjective Cognitive Impairment) [93] (Figure 2), and their verbal recall was tested with the TYM-MCI (Test Your Memory for Mild Cognitive Impairment) [103].

The cognitive abilities of all participants were assessed with the Full-Scale Intelligence Quotient two-subject form (FSIQ-2) of the Wechsler Abbreviated Scale of Intelligence (WASI-II) [104] (Table 1). All participants completed a battery of questionnaires evaluating perceived levels of stress (the Perceived Stress Scale, PSS) [105], sleep quality (the Pittsburgh Sleep Quality Index PSQI) [106], impulsive qualities linked with attention, motor, and non-planning characteristics (the Abbreviated Impulsiveness Scale, ABIS) [107], propensity for risk-taking and sensation-seeking behaviors (the Brief Sensation Seeking Scale, BSSS) [108], anxiety (the Generalized Anxiety Disorder 7-item scale, GAD-7) [109], and depressive symptoms (the Patient Health Questionnaire 9-item scale, PHQ-9) [110]. They provided information about their weekly alcohol intake for the past six months. Average scores and group comparisons for all these measures are presented in Table 1.

### 2.3. ^1^H-MRS and Structural MRI Acquisition

All scans were conducted at the San Diego State University (SDSU) Imaging Center with a 3T Siemens Prisma scanner equipped with a 32-channel head coil. A brief localizer image was initially acquired for each participant to assess the scan quality. Subsequently, high-resolution structural images were obtained with a T1-weighted three-dimensional Magnetization-Prepared Rapid-acquisition Gradient Echo (MPRAGE) sequence with the following parameters: TR = 7.2 ms, TE = 3.01 ms, flip angle = 9°, T1 = 900 ms, inversion repeat time = 2300 ms, bandwidth = 320 Hz/pix, FOV = 256 mm, matrix = 256 × 256, 176 axial slices, GRAPPA = 2, isotropic resolution of 1 mm.

**Figure 3:**
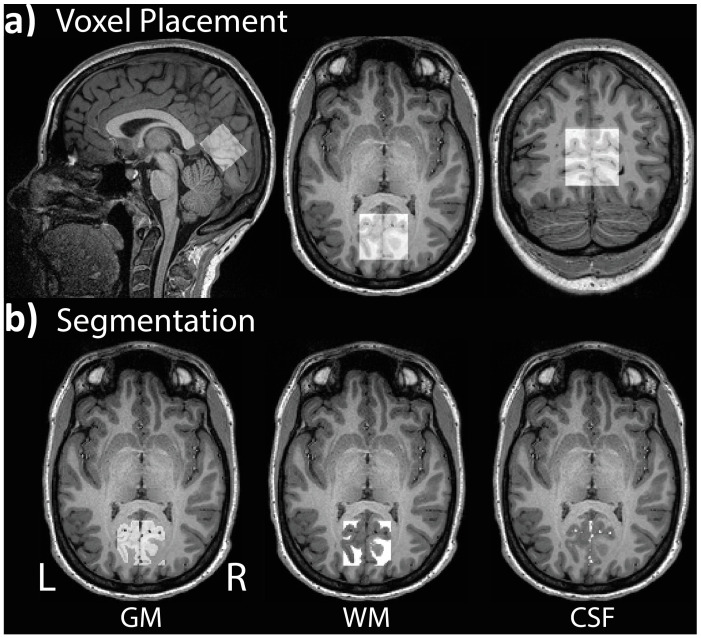
Example of (**a**) voxel placement in the occipital lobe centered on the median and aligned with the tentorium in the sagittal plane; (**b**) segmentation of gray matter (GM), white matter (WM), and cerebrospinal fluid (CSF) for a single participant.

^1^H-MRS spectra were acquired from a voxel located in the occipital lobe. Voxel placement was guided by each participant’s structural scan, with the voxel centered on the median and aligned with the tentorium in the sagittal plane (Figure 3a). Adjustments were made in the axial and coronal planes to ensure that the voxel volume did not include the skull. GABA-edited ^1^H-MRS data were obtained using the Siemens MEGA-PRESS sequence [62,111] from a 30 × 35 × 25 mm (26.3 mL) single voxel of interest (VOI) with the following parameters: TR = 1500 ms, TE = 68 ms, bandwidth = 1670 Hz, 1024 datapoints. A total of 256 averages was collected, including 128 ON and 128 OFF transients, 90° excitation/180° refocusing pulses. The number of acquired signal averages is well within a high SNR range [112]. The bandwidth (full-width half-maximum) of the Gaussian-shaped editing pulses was set to 80 Hz, and the pulses were applied at 1.9 ppm (‘ON’) and 7.5 ppm (‘OFF’) for 128 trials each, with their difference resulting in a J-edited spectrum. The difference signal at 3.0 ppm from the ON/OFF acquisitions contains co-edited contributions from homocarnosine and macromolecules, referred to as GABA+ [53]. Water suppression was accomplished with the Siemens VAPOR full water suppress option. Participants were instructed to close their eyes before the scan began.

One additional participant was scanned, but their GABA+/water value fell 6.6 standard deviations below the group mean, so this data set was excluded from the analysis.

### 2.4. ^1^H-MRS Modeling and Analysis, and VOI Tissue Segmentation

**Figure 4:**
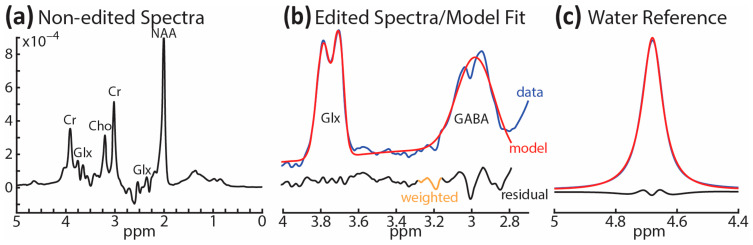
Representative spectra for a single participant: (**a**) non-edited spectra between 0 and 5 ppm; (**b**) edited spectra between 2.8 and 4.2 ppm (blue line) along with fitted peaks showing GABA+ and Glx model (red line) and the residuals (black line); (**c**) reference signal showing the modeling of water from the OFF spectrum.

Modeling and quantification of the ^1^H-MRS data were accomplished with the MATLAB-based (Mathworks, Natick, MA, USA) toolkit Gannet 3.1.3 [113]. For each participant, the following processing steps were applied: spectral registration, frequency and phase correction, 3 Hz exponential line broadening, and rejection of outlier points. ON and OFF spectra were subtracted to generate the edited difference spectrum. Alignment of ON and OFF spectra was accomplished using the total-choline (tCho) peak as a reference signal. A single Gaussian model was used to fit the edited GABA+ signal relative to water (GABA+/w). Fit errors were calculated by dividing the standard deviations of fitting residuals by the fitted GABA+ peak amplitudes. All participants included in the statistical analysis had GABA+/w fit errors ≤ 12% [114,115], with average fit errors equaling 7.2% ± 1.7%. Figure 4 illustrates an example of unedited spectra, the fitted model, and the water reference signal. Two PASC participants reported using Prozac (fluoxetine, a selective serotonin reuptake inhibitor) and Vyvanse (lisdexamfetamine), respectively, but they omitted their medication at least 24 h before the scan. Nonetheless, both of these medications tend to increase GABA levels [85,86,116], which runs counter to the hypothesized GABA decrease, so we checked the two participants’ GABA+/w values. The z-scores were negligibly above the PASC group mean at 0.01 and 0.10, respectively. NAA (N-acetylaspartate) values were determined by calculating the full width at half maximum of the peak at 2 ppm for each participant.

Since tissue composition can influence the spectral quantification, yielding higher GABA+ levels in gray than white matter [117], each participant’s VOI was co-registered to their anatomical scan to ascertain the percentage of gray matter (GM), white matter (WM), and cerebrospinal fluid (CSF) within the volume (Figure 3b). The reported GABA+ concentration values were corrected for CSF-fraction and tissue-dependent relaxation, as recommended by prior publications [118,119]. The average GM, WM, and CSF proportions as well as group comparisons are presented in Table 2. These tissue-dependent segmentation values were used to calculate the GM ratio for each participant based on the following formula: GM/(GM+WM). The GM ratio was applied as a covariate in all analyses of ^1^H-MRS data [117].

### 2.5. Statistical Analysis

Differences in metabolite concentrations between the PASC and CNT groups were tested with one-way ANCOVAs [120] controlling for tissue composition (GM ratio) and biological sex. While alcohol intake did not correlate with GABA+/w (*r* = 0.21, *p* = 0.20), weight-adjusted drinking levels were used as an additional covariate [64]. Associations between ^1^H-MRS metabolites and self-reported measures were evaluated with Pearson’s correlation coefficients. As a special case of structural equation modeling [121,122], mediation analysis was performed to estimate the degree to which a difference in metabolites accounts for (i.e., mediates) the impact of PASC on depression, as measured with the PROMIS scale [91]. The analysis used the PROCESS macro in SPSS and it controlled for all three covariates. Indirect effects were tested with bias-corrected bootstrapping (N = 5000) and 95% confidence intervals for all indices. As shown in the Section 3, mediation analysis estimates the relations between the independent (group) variable and GABA+/w (*a*), GABA+/w and depression (*b*), and PASC and depression (*c’*). The portion of the total effect accounted for by the GABA+/w difference is represented by the product of *a* and *b*, whereas the total effect is expressed as *ab* + *c*’.

## 3. Results

**Figure 5:**
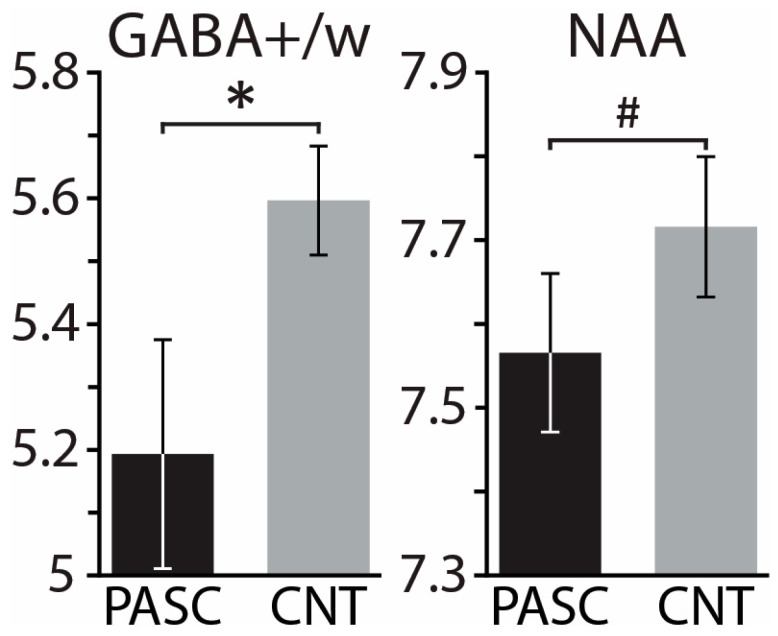
Group means ± standard errors of GABA+/water and NAA (N-acetylaspartate) for PASC and CNT groups. Controlling for the effect of tissue composition, biological sex, and drinking, the PASC group demonstrated lower values than CNT for both metabolites. * *p* < 0.05, # *p* < 0.14.

**Figure 6:**
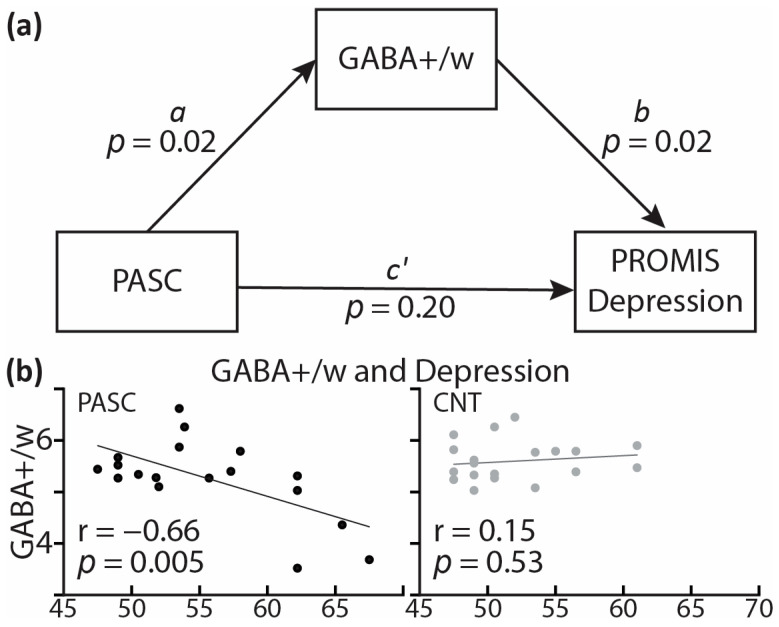
(**a**) Mediation analysis model showing the effect of PASC on depression (PROMIS scale) mediated by GABA+/w. (**b**) Scatterplot indicates that GABA+/w correlates with depression within the PASC group only. Lines represent regression between GABA+/w and depression PROMIS scores on the x-axis. PROMIS: Patient-Reported Outcomes Measurement Information System [91].

Controlling for tissue composition, alcohol intake, and biological sex, the PASC group exhibited lower GABA+/w than CNT, *F*(1,33) = 6.15, *p* = 0.018 (Figure 5). Lower GABA+/w was strongly associated with poor sleep quality, as reflected in the occasional use of sleep-aid medications, as a subscale of the PSQI (*r* = −0.82, *p* = 0.007). Lower GABA+/w was also associated with higher depression, as measured with PROMIS, within the PASC group (*r* = −0.66, *p* = 0.005), but not within the CNT group (*r* = 0.15, *p* = 0.53) (Figure 6b). Including all three covariates, the mediation analysis found that the total impact of PASC on depression resulted in a 4.68 increase in PROMIS t-scores *p* = 0.026, 95% C.I. = [0.60 8.76] relative to the CNT group. Importantly, mediation analysis provides an insight into the indirect effect mediated by GABA+/w as a hypothesized underlying mechanism. The results show that GABA+/w concentration accounted for about 43% of the total effect, *ab* = 2.01, *p* = 0.089, 95% C.I. = [−0.31 4.33], Figure 6a. All parameter estimates, *p*-values, and confidence intervals are presented in Table 3.

The NAA concentration tended to be lower for the PASC group compared to CNT, *F*(1,33) = 2.36, *p* = 0.134, with all three covariates included in the model (Figure 5). Within the PASC group, NAA correlated negatively with verbal recall, *r* = −0.53, *p* = 0.042, tested with the TYM-MCI (Test Your Memory for Mild Cognitive Impairment) [103]. The concentration of Glx/w did not differ between the PASC and CNT groups, *F*(1,33) = 1.07, *p* = 0.309, while controlling for all three covariates.

## 4. Discussion

The PASC syndrome is a health condition marked by a range of sequelae that linger for months or even years after recovery from COVID-19 [3,4,5,6,123,124]. Colloquially termed “long haulers”, people with PASC report a lower quality of life, commonly reflected in neurocognitive deficits, depression, anxiety, fatigue, and insomnia, among other symptoms [5,14,15,16,92,99,125]. Despite a clear need for better understanding of the neural underpinnings of the long-term impact of PASC on these domains, neuroimaging evidence is scant. The present study used ^1^H-MRS to examine the neurochemical profile of the brain tissue in the occipital lobe almost six months after the acute illness in previously healthy adults with PASC in comparison to control participants. The principal results can be summarized as follows: (a) The participants with PASC reported a relative increase in neurocognitive deficits, depression/anxiety, fatigue, and other symptoms (Figure 1), confirming extensive prior evidence. (b) In comparison to the normative scores, the PASC group showed greater cognitive impairment on MISCI [93] and higher levels of anxiety and depression, as assessed by PROMIS [91] (Figure 2). (c) When compared to the locally-recruited CNT group, the PASC participants were matched on demographics, intelligence, and an array of other variables. However, they reported lower sleep quality on PSQI [106] and a higher weekly intake of alcohol (Table 1). (d) Controlling for tissue composition, alcohol intake, and biological sex, the PASC group had lower GABA+/w than CNT in the occipital voxel. (e) Lower GABA+/w levels were associated with poor sleep quality and depression, consistent with the underlying hyperexcitability. (f) Including all three covariates, mediation analysis indicated that the impact of PASC on depression is partly mediated by GABA+/w, suggesting its role as a contributing mechanism. (g) The PASC group tended to have lower NAA levels than CNT.

Participants in this study were mostly young adults who were in good health prior to contracting COVID-19. They recovered from rather mild symptoms and did not require hospitalization. The participants did not experience significant functional limitations in terms of performing their daily duties and activities, as assessed by the Post-COVID-19 Functional Status Scale [87]. However, their overall quality of daily life was moderately impacted by PASC (1.7 ± 1.2 on a scale from 0 to 4). When asked to compare their current level of functioning since recovering from acute COVID-19 on multiple dimensions, the participants with PASC indicated that they experienced the most deterioration in the neurocognitive domain, including greater memory deficits, brain fog, and attention deficits (Figure 1). In addition, they reported higher levels of depression and anxiety, fatigue, loss of smell, insomnia, shortness of breath, headaches, and some other symptoms at lower levels of relative change intensity. None experienced any seizures (Figure 1). Even though our participants reported rather mild PASC symptoms, this profile of relative impairments is consistent with numerous other reports accentuating the importance of the neurocognitive domain, depression/anxiety, fatigue, and insomnia in the overall impact of PASC on the quality of life [9,10,12,14,15,16,17,18,92,99,126,127].

In alignment with a comprehensive body of evidence on PASC-related cognitive deficits, PASC participants scored lower than the normative sample on MISCI (Multidimensional Inventory of Subjective Cognitive Impairment) [93] (Figure 2). Furthermore, their scores for anxiety and depression were higher than in the general population on these PROMIS scales (Patient-Reported Outcomes Measurement Information System) [91] (Figure 2). The PASC group was additionally compared to the control group drawn from the same community and matched on demographic characteristics. As shown in Table 1, the two groups did not differ on general intelligence (WASI-II) [104], stress [105], impulsivity [107], sensation seeking [108], or on brief screening measures of generalized anxiety [109] or depression [110]. However, the PASC participants reported lower sleep quality and more sleep disturbances than the CNT group, as assessed by the Pittsburgh Sleep Quality Index [106]. Reduced sleep quality that persists for months post-infection is one of the principal symptoms associated with PASC, as reported in numerous studies [3,12,34,89,92,97,98,99,126,127]. Insomnia is linked with neural excitability, which increases progressively with the length of waketime in healthy individuals, as shown in studies using electroencephalography (EEG) in combination with transcranial magnetic stimulation (TMS) [128]. Furthermore, TMS studies have confirmed cortical hyperexcitability in people with chronic sleep disturbances [129]. This aligns with ^1^H-MRS reports of lower GABA levels in the occipital cortex and other brain areas in non-medicated people diagnosed with insomnia [130,131]. Indeed, GABA agonists are by far the most commonly prescribed medications for insomnia, since they tip the E/I balance towards inhibition and exert sedative effects, resulting in better sleep [132].

Similarly, depression has been reliably associated with lower GABA levels, as demonstrated using an array of methods including ^1^H-MRS [67,68,70]. Large meta-analyses comparing GABA+ levels across different psychiatric disorders have confirmed that cortical GABA+ is reduced reliably in depressive disorders [61,133]. This evidence has led to the GABAergic deficit hypothesis, proposing that impaired GABAergic neurotransmission underlies the etiology and emergence of depression [134]. This is supported by accumulating evidence from human studies and animal models reporting an E/I imbalance in favor of excitation in depressive disorders, in the context of stress and other moderating factors [135,136]. Conversely, GABA levels increased in the occipital cortex after a two-month-long course of treatment with SSRIs, with particularly notable gains observed in the patients with the lowest GABA levels before treatment [137] and after a course of electroconvulsive therapy [138]. However, cognitive-behavioral therapy did not change levels of GABA+ in the occipital cortex [139], which suggests mechanistic effects of these therapy approaches on the excitation/inhibition balance, as well as the need for patient classification with respect to the sensitivity to treatment based on the relative E/I balance [140].

In the present study, the PASC group had lower GABA+/w than CNT, controlling for tissue composition, alcohol intake, and biological sex (Figure 5). Lower GABA+/w concentration was associated with higher levels of sleep disturbances (*r* = −0.82, *p* = 0.007) and depression (*r* = −0.73, *p* = 0.025). Representative of the core symptoms of PASC, insomnia and depression were correlated in the present study, *r* = 0.40, *p* = 0.01, which is consistent with previous reports [141,142]. The association of lower GABA+/w with increased insomnia and depression is indicative of the underlying neural hyperexcitability. This interpretation is further strengthened by the preliminary results of the mediation analysis indicating that GABA+/w partly mediates the impact of PASC on depression (Figure 6, Table 3). More specifically, as a special case of structural equation modeling, mediation analysis [121,122] indicated that more than 36% of the total impact of PASC on depression could be accounted for by the mediating effect of lower GABA+/w. Though preliminary and in need of replication, this finding is consistent with PASC-related cortical hyperexcitability as an underlying mechanism contributing to depression. Inflammatory activity and immune signaling have been strongly implicated as the underlying mechanisms mediating the risk and emergence of depression [27,35,143,144]. Increased brain inflammation reflected in gliosis is associated with PASC and is particularly prevalent in people with depressive symptoms and/or neurocognitive deficits [33]. Indirect support is additionally provided by the meta-analytical evidence of beneficial effects of SSRI antidepressants on the course of COVID-19 [145] through their anti-inflammatory impact on downregulating cytokine production [146]. 

Indeed, complex interactions between neurotransmission and neuroimmune signaling regulate synaptic plasticity and underlie neural function [147,148]. Thus, neuroinflammation has been proposed as an important aspect of the pathogenesis of PASC-related neurocognitive dysfunction [23,24,25,26,28,29,30,31,32,33,34,35]. Neuroimmune regulatory mechanisms comprise a cascade of processes affecting glutamate and GABA, the two principal neurotransmitters. Increased release and extrasynaptic “spillover” of the excitatory glutamate, along with downregulation of inhibitory GABA, results in neural hyperexcitability [23,149,150]. Neuroinflammatory factors are closely associated with the development of epilepsy [26]. Furthermore, prolonged inflammation has pro-excitatory effects on synaptic activity [24], consistent with the post-illness profile for coronaviruses [28,92] and other conditions eliciting cytokine-mediated response [27]. This results in excessive cortical excitability in people with PASC [25,151,152]. Taken together, these findings confirm that GABA is essential for coordinating and fine-tuning neurotransmission by stabilizing neural networks and the optimal E/I balance [38,54,55,56,57,58,59]. 

In the present study, NAA concentration tended to be lower in the PASC group, controlling for tissue composition, alcohol intake, and biological sex (Figure 5). Previous evidence is limited to only one case–control study, which reported no reliable differences between very small samples of PASC and control participants [74]. However, relatively lower NAA was observed in a single patient with PASC, whose NAA levels improved after three months of memory exercises without medical treatment [153]. Similarly, relatively lower NAA levels were observed in two out of three consecutive patients hospitalized with acute COVID-19 disease [154]. Given that NAA has been used as a marker of compromised neuronal integrity [78,79], lower NAA levels observed in people with PASC may be indicative of neuronal injury or dysfunction. Initially, these changes emerge as a result of the viral infection and are subsequently followed by persisting PASC-related neuroinflammatory processes [155,156]. This is consistent with extensive evidence of reduced NAA levels in people living with HIV, even those with only mild HIV-associated neurocognitive symptoms [83], as well as other viral infections of the nervous system [157]. Furthermore, NAA has been proposed as a biomarker of neuronal recovery since it is sensitive to treatment-reduced reversal. For instance, NAA levels increased after a pharmacological treatment of HIV [158]. Similarly, lower NAA levels have been observed reliably in people diagnosed with depression [70,81,82], which were normalized by SSRI treatment [159,160]. In the present study, the NAA levels were inversely associated with the PASC group scores on a brief test of recent verbal memory [103], which is broadly consistent with reports of moderate correlations with cognitive ability [161]. 

Even though the present study has notable strengths, especially in the context of exceedingly scant evidence on neurochemical alterations associated with PASC, the results should be considered in light of some limitations. The sample size is rather small, which limits the generalizability of the findings, which should be replicated in future research employing larger cohorts. Relatedly, while we controlled for the biological sex variable in our analysis, large-scale studies are needed to clarify potential sex-based interactions with clinical symptom profiles. As is commonly done in ^1^H-MRS studies, the voxel was placed in the occipital cortex to examine the concentration of GABA as an index of cortical neurotransmission in a low-level sensory processing area. However, future studies should investigate possible regional variation in GABA levels.

## 5. Conclusions

Reduced GABA-mediated inhibitory function is indicative of cortical hyperexcitability, contributing to depression, insomnia, and other PASC symptoms. In addition, marginally lower NAA is suggestive of compromised neuronal integrity. While preliminary, these findings are consistent with persistent neuroinflammation as an important aspect of the pathogenesis of PASC-related neurocognitive deficits and affective distress.

## Figures and Tables

**Table 1:** Participant characteristics for PASC and CNT groups.

	PASC (*n* = 18)	CNT (*n* = 20)	F(1,36)/χ^2^	*p*
% Women	61%	60%	0.01 ^a^	0.944
% White/Non-Hispanic	83%	50%	5.38 ^a^	0.146
Age	24.44 ± 5.25	23.35 ± 3.66	0.57	0.457
Intelligence (WASI)	109.00 ± 12.50	111.30 ± 9.03	0.87	0.359
Stress (PSS)	19.56 ± 4.57	18.80 ± 4.77	0.25	0.622
Sleep Quality Total (PSQI)	9.22 ± 3.52	5.70 ± 2.39	13.25	**<0.001**
Impulsivity (ABIS)				
Attention	1.73 ± 0.30	1.76 ± 0.41	0.06	0.813
Motor	1.77 ± 0.37	1.60 ± 0.41	1.36	0.253
Non-Planning	1.97 ± 0.48	1.80 ± 0.57	0.72	0.404
Sensation Seeking (BSSS)				
Experience	7.38 ± 1.28	7.65 ± 1.84	0.19	0.667
Boredom	6.09 ± 1.30	6.35 ± 1.84	0.17	0.683
Thrill	6.74 ± 1.87	6.30 ± 2.11	0.34	0.567
Disinhibition	5.60 ± 1.85	5.55 ± 1.79	0.01	0.942
Anxiety (GAD-7)	4.50 ± 3.13	3.28 ± 3.01	1.43	0.240
Depression (PHQ-9)	4.33 ± 3.45	2.80 ± 2.89	2.22	0.145
Avg Drinks/Week	4.45 ± 4.87	1.69 ± 1.46	5.88	**0.020**
Memory (TYM-MCI)	9.60 ± 3.02			

Group means ± standard deviations were calculated for all continuous variables. Group differences were analyzed with one-way ANOVAs. Categorical variables (sex and ethnicity) are presented as percentages. Group differences that were evaluated with the Χ^2^ test are marked with ^a^. Statistically significant *p*-values are marked in bold. WASI: Weschler Abbreviated Scale of Intelligence, PSS: Perceived Stress Scale, PSQI: Pittsburgh Sleep Quality Index, ABIS: Abbreviated Impulsiveness Scale, BSSS: Brief Sensation Seeking Scale, GAD-7: Generalized Anxiety Disorder, PHQ-9: Patient Health Questionnaire (depression), TYM-MCI: Test Your Memory for Mild Cognitive Impairment.

**Table 2:** Tissue segmentation.

VOI	PASC (*n* = 18)	CNT (*n* = 20)	F(1,36)	*p*
GM	0.63 ± 0.05	0.62 ± 0.03	0.69	0.413
WM	0.29 ± 0.03	0.29 ± 0.03	0.84	0.366
CSF	0.09 ± 0.03	0.09 ± 0.02	0.10	0.760

Group means ± standard deviations for tissue segmentation values for gray matter (GM), white matter (WM), and cerebrospinal fluid (CSF) in the occipital voxel for the PASC and CNT groups.

**Table 3:** Mediation parameter estimates.

Parameter	Est.	*p*	*95% C.I.*
a	−0.51	0.017	[−0.92 −0.10]
b	−3.94	0.023	[−7.31 −0.58]
c’	2.67	0.202	[−1.51 6.85]
ab	2.01	0.089	[−0.31 4.33]
ab + c’	4.68	0.026	[0.60 8.76]

## Data Availability

Data are available at the following link: https://stbil.sdsu.edu/data/marinkovic_2023_data.xlsx.

## References

[1] Cutler D.M., Summers L.H. (2020). The COVID-19 Pandemic and the $16 Trillion Virus. JAMA.

[2] Smith M.P. (2022). Estimating total morbidity burden of COVID-19: Relative importance of death and disability. J. Clin. Epidemiol..

[3] CDC Long COVID or Post-COVID Conditions. https://www.cdc.gov/coronavirus/2019-ncov/long-term-effects/index.html.

[4] DHHS (2022). National Research Action Plan on Long COVID.

[5] Zadeh F.H., Wilson D.R., Agrawal D.K. (2023). Long COVID: Complications, Underlying Mechanisms, and Treatment Strategies. Arch. Microbiol. Immunol..

[6] Soriano J.B., Murthy S., Marshall J.C., Relan P., Diaz J.V. (2022). A clinical case definition of post-COVID-19 condition by a Delphi consensus. Lancet Infect. Dis..

[7] Kubota T., Kuroda N., Sone D. (2023). Neuropsychiatric aspects of long COVID: A comprehensive review. Psychiatry Clin. Neurosci..

[8] Groff D., Sun A., Ssentongo A.E., Ba D.M., Parsons N., Poudel G.R., Lekoubou A., Oh J.S., Ericson J.E., Ssentongo P. (2021). Short-term and long-term rates of postacute sequelae of SARS-CoV-2 infection: A systematic review. JAMA Netw. Open.

[9] Thaweethai T., Jolley S.E., Karlson E.W., Levitan E.B., Levy B., McComsey G.A., McCorkell L., Nadkarni G.N., Parthasarathy S., Singh U. (2023). Development of a Definition of Postacute Sequelae of SARS-CoV-2 Infection. JAMA.

[10] Henneghan A.M., Lewis K.A., Gill E., Kesler S.R. (2022). Cognitive impairment in non-critical, mild-to-moderate COVID-19 survivors. Front. Psychol..

[11] Schild A.-K., Goereci Y., Scharfenberg D., Klein K., Lülling J., Meiberth D., Schweitzer F., Stürmer S., Zeyen P., Sahin D. (2023). Multidomain cognitive impairment in non-hospitalized patients with the post-COVID-19 syndrome: Results from a prospective monocentric cohort. J. Neurol..

[12] Munipalli B., Seim L., Dawson N.L., Knight D., Dabrh A.M.A. (2022). Post-acute sequelae of COVID-19 (PASC): A meta-narrative review of pathophysiology, prevalence, and management. SN Compr. Clin. Med..

[13] Bowe B., Xie Y., Al-Aly Z. (2023). Postacute sequelae of COVID-19 at 2 years. Nat. Med..

[14] Nalbandian A., Sehgal K., Gupta A., Madhavan M.V., McGroder C., Stevens J.S., Cook J.R., Nordvig A.S., Shalev D., Sehrawat T.S. (2021). Post-acute COVID-19 syndrome. Nat. Med..

[15] Davis H.E., Assaf G.S., McCorkell L., Wei H., Low R.J., Re’em Y., Redfield S., Austin J.P., Akrami A. (2021). Characterizing long COVID in an international cohort: 7 months of symptoms and their impact. EClinicalMedicine.

[16] Graham E.L., Clark J.R., Orban Z.S., Lim P.H., Szymanski A.L., Taylor C., DiBiase R.M., Jia D.T., Balabanov R., Ho S.U. (2021). Persistent neurologic symptoms and cognitive dysfunction in non-hospitalized COVID-19 “long haulers”. Ann. Clin. Transl. Neurol..

[17] Kondratiuk A.L., Pillay T.D., Kon O.M., Lalvani A. (2021). A conceptual framework to accelerate the clinical impact of evolving research into long COVID. Lancet Infect. Dis..

[18] Komaroff A.L., Bateman L. (2020). Will COVID-19 Lead to Myalgic Encephalomyelitis/Chronic Fatigue Syndrome?. Front. Med..

[19] Bougakov D., Podell K., Goldberg E. (2021). Multiple Neuroinvasive Pathways in COVID-19. Mol. Neurobiol..

[20] Yan R., Zhang Y., Li Y., Xia L., Guo Y., Zhou Q. (2020). Structural basis for the recognition of SARS-CoV-2 by full-length human ACE2. Science.

[21] Baig A.M., Khaleeq A., Ali U., Syeda H. (2020). Evidence of the COVID-19 Virus Targeting the CNS: Tissue Distribution, Host-Virus Interaction, and Proposed Neurotropic Mechanisms. ACS Chem. Neurosci..

[22] Iadecola C., Anrather J., Kamel H. (2020). Effects of COVID-19 on the Nervous System. Cell.

[23] Haroon E., Miller A.H., Sanacora G. (2017). Inflammation, Glutamate, and Glia: A Trio of Trouble in Mood Disorders. Neuropsychopharmacology.

[24] Maggio N., Shavit-Stein E., Dori A., Blatt I., Chapman J. (2013). Prolonged systemic inflammation persistently modifies synaptic plasticity in the hippocampus: Modulation by the stress hormones. Front. Mol. Neurosci..

[25] Manganotti P., Michelutti M., Furlanis G., Deodato M., Stella A.B. (2023). Deficient GABABergic and glutamatergic excitability in the motor cortex of patients with long-COVID and cognitive impairment. Clin. Neurophysiol..

[26] Paudel Y.N., Shaikh M.F., Shah S., Kumari Y., Othman I. (2018). Role of inflammation in epilepsy and neurobehavioral comorbidities: Implication for therapy. Eur. J. Pharmacol..

[27] Dantzer R., O’Connor J.C., Freund G.G., Johnson R.W., Kelley K.W. (2008). From inflammation to sickness and depression: When the immune system subjugates the brain. Nat. Rev. Neurosci..

[28] Mondelli V., Pariante C.M. (2021). What can neuroimmunology teach us about the symptoms of long-COVID?. Oxf. Open Immunol..

[29] Heneka M., Golenbock D., Latz E., Morgan D., Brown R. (2020). Immediate and long-term consequences of COVID-19 infections for the development of neurological disease. Alzheimers Res. Ther..

[30] Goldberg E., Podell K., Sodickson D.K., Fieremans E. (2021). The brain after COVID-19: Compensatory neurogenesis or persistent neuroinflammation?. EClinicalMedicine.

[31] Mazza M.G., Palladini M., De Lorenzo R., Magnaghi C., Poletti S., Furlan R., Ciceri F., Rovere-Querini P., Benedetti F., COVID-19 BioB Outpatient Clinic Study Group (2021). Persistent psychopathology and neurocognitive impairment in COVID-19 survivors: Effect of inflammatory biomarkers at three-month follow-up. Brain Behav. Immun..

[32] Loftis J.M., Firsick E., Shirley K., Adkins J.L., Le-Cook A., Sano E., Hudson R., Moorman J. (2023). Inflammatory and mental health sequelae of COVID-19. Compr. Psychoneuroendocrinology.

[33] Braga J., Lepra M., Kish S.J., Rusjan P.M., Nasser Z., Verhoeff N., Vasdev N., Bagby M., Boileau I., Husain M.I. (2023). Neuroinflammation After COVID-19 With Persistent Depressive and Cognitive Symptoms. JAMA Psychiatry.

[34] Li Z., Zhang Z., Zhang Z., Wang Z., Li H. (2023). Cognitive impairment after long COVID-19: Current evidence and perspectives. Front. Neurol..

[35] Al-Hakeim H.K., Al-Rubaye H.T., Al-Hadrawi D.S., Almulla A.F., Maes M. (2023). Long-COVID post-viral chronic fatigue and affective symptoms are associated with oxidative damage, lowered antioxidant defenses and inflammation: A proof of concept and mechanism study. Mol. Psychiatry.

[36] Bullmore E., Sporns O. (2012). The economy of brain network organization. Nat. Rev. Neurosci..

[37] Amzica F., Lopes da Silva F.H., Schomer D., Lopes da Silva F.H. (2011). Cellular substrates of brain rhythms. Niedermeyer’s Electroencephalography: Basic Principles, Clinical Applications, and Related Fields.

[38] Buzsaki G. (2006). Rhythms of the Brain.

[39] Knight R.T. (2007). Neuroscience. Neural networks debunk phrenology. Science.

[40] Haider B., Duque A., Hasenstaub A.R., McCormick D.A. (2006). Neocortical network activity in vivo is generated through a dynamic balance of excitation and inhibition. J. Neurosci..

[41] Tatti R., Haley M.S., Swanson O.K., Tselha T., Maffei A. (2017). Neurophysiology and Regulation of the Balance Between Excitation and Inhibition in Neocortical Circuits. Biol. Psychiatry.

[42] Rubenstein J.L., Merzenich M.M. (2003). Model of autism: Increased ratio of excitation/inhibition in key neural systems. Genes. Brain Behav..

[43] Sohal V.S., Rubenstein J.L. (2019). Excitation-inhibition balance as a framework for investigating mechanisms in neuropsychiatric disorders. Mol. Psychiatry.

[44] Selten M., van Bokhoven H., Nadif Kasri N. (2018). Inhibitory control of the excitatory/inhibitory balance in psychiatric disorders. F1000Res.

[45] Gao R., Penzes P. (2015). Common mechanisms of excitatory and inhibitory imbalance in schizophrenia and autism spectrum disorders. Curr. Mol. Med..

[46] Marin O. (2012). Interneuron dysfunction in psychiatric disorders. Nat. Rev. Neurosci..

[47] Nunez P.L., Srinivasan R. (2006). Electric Fields of the Brain: The Neurophysics of EEG.

[48] Prichard J.W., Shulman R.G. (1986). NMR spectroscopy of brain metabolism in vivo. Annu. Rev. Neurosci..

[49] Radda G.K., Rajagopalan B., Taylor D.J. (1989). Biochemistry in vivo: An appraisal of clinical magnetic resonance spectroscopy. Magn. Reson. Q..

[50] Bluml S., Bluml S., Panigrahy A. (2012). Magnetic resonance spectroscopy: Basics. MR Spectroscopy of Pediatric Brain Disorders.

[51] Cox I.J. (1996). Development and applications of in vivo clinical magnetic resonance spectroscopy. Prog. Biophys. Mol. Biol..

[52] Ende G. (2015). Proton Magnetic Resonance Spectroscopy: Relevance of Glutamate and GABA to Neuropsychology. Neuropsychol. Rev..

[53] Harris A.D., Saleh M.G., Edden R.A. (2017). Edited (1) H magnetic resonance spectroscopy in vivo: Methods and metabolites. Magn. Reson. Med..

[54] Koh W., Kwak H., Cheong E., Lee C.J. (2023). GABA tone regulation and its cognitive functions in the brain. Nat. Rev. Neurosci..

[55] Tremblay R., Lee S., Rudy B. (2016). GABAergic interneurons in the neocortex: From cellular properties to circuits. Neuron.

[56] Markram H., Toledo-Rodriguez M., Wang Y., Gupta A., Silberberg G., Wu C. (2004). Interneurons of the neocortical inhibitory system. Nat. Rev. Neurosci..

[57] Roux L., Buzsaki G. (2015). Tasks for inhibitory interneurons in intact brain circuits. Neuropharmacology.

[58] DeFelipe J., Alonso-Nanclares L., Arellano J.I. (2002). Microstructure of the neocortex: Comparative aspects. J. Neurocytol..

[59] Kolasinski J., Logan J.P., Hinson E.L., Manners D., Zand A.P.D., Makin T.R., Emir U.E., Stagg C.J. (2017). A mechanistic link from GABA to cortical architecture and perception. Curr. Biol..

[60] Yizhar O., Fenno L.E., Prigge M., Schneider F., Davidson T.J., O’Shea D.J., Sohal V.S., Goshen I., Finkelstein J., Paz J.T. (2011). Neocortical excitation/inhibition balance in information processing and social dysfunction. Nature.

[61] Schur R.R., Draisma L.W., Wijnen J.P., Boks M.P., Koevoets M.G., Joels M., Klomp D.W., Kahn R.S., Vinkers C.H. (2016). Brain GABA levels across psychiatric disorders: A systematic literature review and meta-analysis of (1) H-MRS studies. Hum. Brain Mapp..

[62] Mescher M., Merkle H., Kirsch J., Garwood M., Gruetter R. (1998). Simultaneous in vivo spectral editing and water suppression. NMR Biomed..

[63] Petroff O.A., Hyder F., Rothman D.L., Mattson R.H. (2001). Homocarnosine and seizure control in juvenile myoclonic epilepsy and complex partial seizures. Neurology.

[64] Marinkovic K., Alderson Myers A.B., Arienzo D., Sereno M.I., Mason G.F. (2022). Cortical GABA levels are reduced in young adult binge drinkers: Association with recent alcohol consumption and sex. Neuroimage Clin..

[65] Rae C.D. (2014). A guide to the metabolic pathways and function of metabolites observed in human brain 1H magnetic resonance spectra. Neurochem. Res..

[66] Saleh M.G., Chang L., Liang H., Ryan M.C., Cunningham E., Garner J., Wilson E., Levine A.R., Kottilil S., Ernst T. (2023). Ongoing oxidative stress in individuals with post-acute sequelae of COVID-19. Neuroimmune Pharmacol. Ther..

[67] Sanacora G., Mason G.F., Krystal J.H. (2000). Impairment of GABAergic transmission in depression: New insights from neuroimaging studies. Crit. Rev. Neurobiol..

[68] Sanacora G., Gueorguieva R., Epperson C.N., Wu Y.-T., Appel M., Rothman D.L., Krystal J.H., Mason G.F. (2004). Subtype-specific alterations of γ-aminobutyric acid and glutamatein patients with major depression. Arch. Gen. Psychiat..

[69] Hasler G., van der Veen J.W., Tumonis T., Meyers N., Shen J., Drevets W.C. (2007). Reduced prefrontal glutamate/glutamine and γ-aminobutyric acid levels in major depression determined using proton magnetic resonance spectroscopy. Arch. Gen. Psychiat..

[70] Bhagwagar Z., Wylezinska M., Jezzard P., Evans J., Ashworth F., Sule A., Matthews P.M., Cowen P.J. (2007). Reduction in occipital cortex γ-aminobutyric acid concentrations in medication-free recovered unipolar depressed and bipolar subjects. Biol. Psychiat..

[71] Price R.B., Shungu D.C., Mao X., Nestadt P., Kelly C., Collins K.A., Murrough J.W., Charney D.S., Mathew S.J. (2009). Amino acid neurotransmitters assessed by 1H MRS: Relationship to treatment-resistance in major depressive disorder. Biol. Psychiat..

[72] Crowley T., Cryan J.F., Downer E.J., O’Leary O.F. (2016). Inhibiting neuroinflammation: The role and therapeutic potential of GABA in neuro-immune interactions. Brain Behav. Immun..

[73] Tian J., Kaufman D.L. (2023). The GABA and GABA-Receptor System in Inflammation, Anti-Tumor Immune Responses, and COVID-19. Biomedicines.

[74] Sklinda K., Górecki A., Dorobek M., Walecki J., Modrzyńska A., Mruk B. (2021). Ischaemic background of brain fog in long haul COVID-19–a nuclear magnetic resonance spectroscopy-based metabonomic analysis. Preliminary results. Pol. J. Radiol..

[75] Ramadan S., Lin A., Stanwell P. (2013). Glutamate and glutamine: A review of in vivo MRS in the human brain. NMR Biomed..

[76] Inglese M., Rusinek H., George I.C., Babb J.S., Grossman R.I., Gonen O. (2008). Global average gray and white matter N-acetylaspartate concentration in the human brain. Neuroimage.

[77] Urenjak J., Williams S.R., Gadian D.G., Noble M. (1993). Proton nuclear magnetic resonance spectroscopy unambiguously identifies different neural cell types. J. Neurosci..

[78] Moffett J.R., Ross B., Arun P., Madhavarao C.N., Namboodiri A.M. (2007). N-Acetylaspartate in the CNS: From neurodiagnostics to neurobiology. Prog. Neurobiol..

[79] Sullivan E.V., Adalsteinsson E., Spielman D.M., Hurd R.E., Pfefferbaum A. (2001). N-acetylaspartate—A marker of neuronal integrity. Ann. Neurol..

[80] Joyce J.M., La P.L., Walker R., Harris A.D. (2022). Magnetic resonance spectroscopy of traumatic brain injury and subconcussive hits: A systematic review and meta–analysis. J. Neurotrauma.

[81] Kong L., Li H., Lin F., Zheng W., Zhang H., Wu R. (2023). Neurochemical and microstructural alterations in bipolar and depressive disorders: A multimodal magnetic resonance imaging study. Front. Neurol..

[82] Li H., Xu H., Zhang Y., Guan J., Zhang J., Xu C., Shen Z., Xiao B., Liang C., Chen K. (2016). Differential neurometabolite alterations in brains of medication-free individuals with bipolar disorder and those with unipolar depression: A two-dimensional proton magnetic resonance spectroscopy study. Bipolar Disord..

[83] Chang L., Munsaka S.M., Kraft-Terry S., Ernst T. (2013). Magnetic resonance spectroscopy to assess neuroinflammation and neuropathic pain. J. Neuroimmune Pharmacol..

[84] Paslakis G., Träber F., Roberz J., Block W., Jessen F. (2014). N-acetyl-aspartate (NAA) as a correlate of pharmacological treatment in psychiatric disorders: A systematic review. Eur. Neuropsychopharmacol..

[85] Robinson R.T., Drafts B.C., Fisher J.L. (2003). Fluoxetine increases GABAA receptor activity through a novel modulatory site. J. Pharmacol. Exp. Ther..

[86] Pereira F.C., Rolo M.R., Marques E., Mendes V.M., Ribeiro C.F., Ali S.F., Morgadinho T., Macedo T.R. (2008). Acute increase of the glutamate–glutamine cycling in discrete brain areas after administration of a single dose of amphetamine. Ann. N. Y. Acad. Sci..

[87] Klok F.A., Boon G., Barco S., Endres M., Geelhoed J.J.M., Knauss S., Rezek S.A., Spruit M.A., Vehreschild J., Siegerink B. (2020). The Post-COVID-19 Functional Status scale: A tool to measure functional status over time after COVID-19. Eur. Respir. J..

[88] Premraj L., Kannapadi N.V., Briggs J., Seal S.M., Battaglini D., Fanning J., Suen J., Robba C., Fraser J., Cho S.-M. (2022). Mid and long-term neurological and neuropsychiatric manifestations of post-COVID-19 syndrome: A meta-analysis. J. Neurol. Sci..

[89] Han Q., Zheng B., Daines L., Sheikh A. (2022). Long-term sequelae of COVID-19: A systematic review and meta-analysis of one-year follow-up studies on post-COVID symptoms. Pathogens.

[90] Jennings G., Monaghan A., Xue F., Mockler D., Romero-Ortuño R. (2021). A systematic review of persistent symptoms and residual abnormal functioning following acute COVID-19: Ongoing symptomatic phase vs. post-COVID-19 syndrome. J. Clin. Med..

[91] Carle A.C., Riley W., Hays R.D., Cella D. (2015). Confirmatory Factor Analysis of the Patient Reported Outcomes Measurement Information System (PROMIS) Adult Domain Framework Using Item Response Theory Scores. Med. Care.

[92] Rogers J.P., Chesney E., Oliver D., Pollak T.A., McGuire P., Fusar-Poli P., Zandi M.S., Lewis G., David A.S. (2020). Psychiatric and neuropsychiatric presentations associated with severe coronavirus infections: A systematic review and meta-analysis with comparison to the COVID-19 pandemic. Lancet Psychiatry.

[93] Kratz A.L., Schilling S.G., Goesling J., Williams D.A. (2015). Development and initial validation of a brief self-report measure of cognitive dysfunction in fibromyalgia. J. Pain.

[94] Rubega M., Ciringione L., Bertuccelli M., Paramento M., Sparacino G., Vianello A., Masiero S., Vallesi A., Formaggio E., Del Felice A. (2022). High-density EEG sleep correlates of cognitive and affective impairment at 12-month follow-up after COVID-19. Clin. Neurophysiol..

[95] Saucier J., Jose C., Beroual Z., Al-Qadi M., Chartrand S., Libert E., Losier M.-C., Cooling K., Girouard G., Jbilou J. (2023). Cognitive inhibition deficit in long COVID-19: An exploratory study. Front. Neurol..

[96] Rothrock N., Amtmann D., Cook K. (2020). Development and validation of an interpretive guide for PROMIS scores. J. Patient Rep. Outcomes.

[97] Percze A.R., Nagy A., Polivka L., Barczi E., Czaller I., Kovats Z., Varga J.T., Ballai J.H., Muller V., Horvath G. (2023). Fatigue, sleepiness and sleep quality are SARS-CoV-2 variant independent in patients with long COVID symptoms. Inflammopharmacology.

[98] Nowakowski S., Kokonda M., Sultana R., Duong B.B., Nagy S.E., Zaidan M.F., Baig M.M., Grigg B.V., Seashore J., Deer R.R. (2022). Association between sleep quality and mental health among patients at a post-COVID-19 recovery clinic. Brain Sci..

[99] Lauria A., Carfì A., Benvenuto F., Bramato G., Ciciarello F., Rocchi S., Rota E., Salerno A., Stella L., Tritto M. (2023). Neuropsychological measures of post-COVID-19 cognitive status. Front. Psychol..

[100] Kerr W.C., Ye Y., Martinez P., Karriker-Jaffe K.J., Patterson D., Greenfield T.K., Mulia N. (2022). Longitudinal assessment of drinking changes during the pandemic: The 2021 COVID-19 follow-up study to the 2019 to 2020 National Alcohol Survey. Alcohol. Clin. Exp. Res..

[101] Castaldelli-Maia J.M., Segura L.E., Martins S.S. (2021). The concerning increasing trend of alcohol beverage sales in the US during the COVID-19 pandemic. Alcohol.

[102] QualtricsXM. https://www.qualtrics.com/.

[103] Brown J.M., Lansdall C.J., Wiggins J., Dawson K.E., Hunter K., Rowe J.B., Parker R.A. (2017). The Test Your Memory for Mild Cognitive Impairment (TYM-MCI). J. Neurol. Neurosurg. Psychiatry.

[104] Wechsler D. (1999). Wechsler Abbreviated Scale of Intelligence (WASI—II).

[105] Cohen S., Kamarck T., Mermelstein R. (1983). A global measure of perceived stress. J. Health Soc. Behav..

[106] Buysse D.J., Reynolds C.F., Monk T.H., Berman S.R., Kupfer D.J. (1989). The Pittsburgh Sleep Quality Index: A new instrument for psychiatric practice and research. Psychiatry Res..

[107] Coutlee C.G., Politzer C.S., Hoyle R.H., Huettel S.A. (2014). An abbreviated impulsiveness scale constructed through confirmatory factor analysis of the Barratt Impulsiveness Scale version 11. Arch. Sci. Psychol..

[108] Hoyle R.H., Stephenson M.T., Palmgreen P., Lorch E.P., Donohew R.L. (2002). Reliability and validity of a brief measure of sensation seeking. Pers. Indiv. Differ..

[109] Spitzer R.L., Kroenke K., Williams J.B., Lowe B. (2006). A brief measure for assessing generalized anxiety disorder: The GAD-7. Arch. Intern. Med..

[110] Kroenke K., Spitzer R.L. (2002). The PHQ-9: A new depression diagnostic and severity measure. Psychiatr. Ann..

[111] Mullins P.G., Chen H., Xu J., Caprihan A., Gasparovic C. (2008). Comparative reliability of proton spectroscopy techniques designed to improve detection of J-coupled metabolites. Magn. Reson. Med..

[112] Mikkelsen M., Loo R.S., Puts N.A.J., Edden R.A.E., Harris A.D. (2018). Designing GABA-edited magnetic resonance spectroscopy studies: Considerations of scan duration, signal-to-noise ratio and sample size. J. Neurosci. Methods.

[113] Edden R.A., Puts N.A., Harris A.D., Barker P.B., Evans C.J. (2014). Gannet: A batch-processing tool for the quantitative analysis of gamma-aminobutyric acid-edited MR spectroscopy spectra. J. Magn. Reson. Imaging.

[114] Cuypers K., Hehl M., van Aalst J., Chalavi S., Mikkelsen M., Van Laere K., Dupont P., Mantini D., Swinnen S.P. (2021). Age-related GABAergic differences in the primary sensorimotor cortex: A multimodal approach combining PET, MRS and TMS. Neuroimage.

[115] Puts N.A.J., Heba S., Harris A.D., Evans C.J., McGonigle D.J., Tegenthoff M., Schmidt-Wilcke T., Edden R.A.E. (2018). GABA Levels in Left and Right Sensorimotor Cortex Correlate across Individuals. Biomedicines.

[116] Bhagwagar Z., Wylezinska M., Taylor M., Jezzard P., Matthews P.M., Cowen P.J. (2004). Increased brain GABA concentrations following acute administration of a selective serotonin reuptake inhibitor. Am. J. Psychiatry.

[117] Peek A., Rebbeck T., Leaver A., Foster S.L., Refshauge K., Puts N., Oeltzschner G., Andronesi O.C., Barker P.B., Bogner W. (2023). A comprehensive guide to MEGA-PRESS for GABA measurement. Anal. Biochem..

[118] Gasparovic C., Song T., Devier D., Bockholt H.J., Caprihan A., Mullins P.G., Posse S., Jung R.E., Morrison L.A. (2006). Use of tissue water as a concentration reference for proton spectroscopic imaging. Magn. Reson. Med..

[119] Mullins P.G., McGonigle D.J., O’Gorman R.L., Puts N.A., Vidyasagar R., Evans C.J., Edden R.A., Cardiff Symposium on MRS of GABA (2014). Current practice in the use of MEGA-PRESS spectroscopy for the detection of GABA. Neuroimage.

[120] SPSS (2017). IBM SPSS Statistics for Windows.

[121] MacKinnon D.P., Fairchild A.J., Fritz M.S. (2007). Mediation analysis. Annu. Rev. Psychol..

[122] Hayes A.F. (2009). Beyond Baron and Kenny: Statistical mediation analysis in the new millennium. Commun. Monogr..

[123] Cheetham N.J., Penfold R., Giunchiglia V., Bowyer V., Sudre C.H., Canas L.S., Deng J., Murray B., Kerfoot E., Antonelli M. (2023). The effects of COVID-19 on cognitive performance in a community-based cohort: A COVID symptom study biobank prospective cohort study. EClinicalMedicine.

[124] Du Y., Zhao W., Huang S., Huang Y., Chen Y., Zhang H., Guo H., Liu J. (2023). Two-year follow-up of brain structural changes in patients who recovered from COVID-19: A prospective study. Psychiatry Res..

[125] Nouraeinejad A. (2022). Brain fog as a Long-term Sequela of COVID-19. SN Compr. Clin. Med..

[126] Stefanou M.-I., Palaiodimou L., Bakola E., Smyrnis N., Papadopoulou M., Paraskevas G.P., Rizos E., Boutati E., Grigoriadis N., Krogias C. (2022). Neurological manifestations of long-COVID syndrome: A narrative review. Ther. Adv. Chronic Dis..

[127] Taquet M., Geddes J.R., Husain M., Luciano S., Harrison P.J. (2021). 6-month neurological and psychiatric outcomes in 236 379 survivors of COVID-19: A retrospective cohort study using electronic health records. Lancet Psychiatry.

[128] Huber R., Mäki H., Rosanova M., Casarotto S., Canali P., Casali A.G., Tononi G., Massimini M. (2013). Human cortical excitability increases with time awake. Cereb. Cortex.

[129] Lanza G., Cantone M., Lanuzza B., Pennisi M., Bella R., Pennisi G., Ferri R. (2015). Distinctive patterns of cortical excitability to transcranial magnetic stimulation in obstructive sleep apnea syndrome, restless legs syndrome, insomnia, and sleep deprivation. Sleep Med. Rev..

[130] Plante D.T., Jensen J.E., Schoerning L., Winkelman J.W. (2012). Reduced γ-aminobutyric acid in occipital and anterior cingulate cortices in primary insomnia: A link to major depressive disorder?. Neuropsychopharmacology.

[131] Winkelman J.W., Buxton O.M., Jensen J.E., Benson K.L., O’Connor S.P., Wang W., Renshaw P.F. (2008). Reduced brain GABA in primary insomnia: Preliminary data from 4T proton magnetic resonance spectroscopy (1H-MRS). Sleep.

[132] Roth T. (2007). A physiologic basis for the evolution of pharmacotherapy for insomnia. J. Clin. Psychiatry.

[133] Godfrey K.E., Gardner A.C., Kwon S., Chea W., Muthukumaraswamy S.D. (2018). Differences in excitatory and inhibitory neurotransmitter levels between depressed patients and healthy controls: A systematic review and meta-analysis. J. Psychiatr. Res..

[134] Luscher B., Fuchs T. (2015). GABAergic control of depression-related brain states. Advances in Pharmacology.

[135] Fogaça M.V., Duman R.S. (2019). Cortical GABAergic dysfunction in stress and depression: New insights for therapeutic interventions. Front. Cell. Neurosci..

[136] Cutler A.J., Mattingly G.W., Maletic V. (2023). Understanding the mechanism of action and clinical effects of neuroactive steroids and GABAergic compounds in major depressive disorder. Transl. Psychiatry.

[137] Sanacora G., Mason G.F., Rothman D.L., Krystal J.H. (2002). Increased occipital cortex GABA concentrations in depressed patients after therapy with selective serotonin reuptake inhibitors. Am. J. Psychiatry.

[138] Sanacora G., Mason G.F., Rothman D.L., Hyder F., Ciarcia J.J., Ostroff R.B., Berman R.M., Krystal J.H. (2003). Increased cortical GABA concentrations in depressed patients receiving ECT. Am. J. Psychiatry.

[139] Abdallah C.G., Niciu M.J., Fenton L.R., Fasula M.K., Jiang L., Black A., Rothman D.L., Mason G.F., Sanacora G. (2014). Decreased occipital cortical glutamate levels in response to successful cognitive-behavioral therapy and pharmacotherapy for major depressive disorder. Psychother. Psychosom..

[140] Caverzasi E., Pichiecchio A., Poloni G.U., Calligaro A., Pasin M., Palesi F., Castellazzi G., Pasquini M., Biondi M., Barale F. (2012). Magnetic resonance spectroscopy in the evaluation of treatment efficacy in unipolar major depressive disorder: A review of the literature. Funct. Neurol..

[141] Nutt D., Wilson S., Paterson L. (2022). Sleep disorders as core symptoms of depression. Dialogues Clin. Neurosci..

[142] Mollayeva T., Thurairajah P., Burton K., Mollayeva S., Shapiro C.M., Colantonio A. (2016). The Pittsburgh sleep quality index as a screening tool for sleep dysfunction in clinical and non-clinical samples: A systematic review and meta-analysis. Sleep Med. Rev..

[143] Berk M., Williams L.J., Jacka F.N., O’Neil A., Pasco J.A., Moylan S., Allen N.B., Stuart A.L., Hayley A.C., Byrne M.L. (2013). So depression is an inflammatory disease, but where does the inflammation come from?. BMC Med..

[144] Roohi E., Jaafari N., Hashemian F. (2021). On inflammatory hypothesis of depression: What is the role of IL-6 in the middle of the chaos?. J. Neuroinflammation.

[145] Nakhaee H., Zangiabadian M., Bayati R., Rahmanian M., Ghaffari Jolfayi A., Rakhshanderou S. (2022). The effect of antidepressants on the severity of COVID-19 in hospitalized patients: A systematic review and meta-analysis. PLoS ONE.

[146] Lu Y., Ho C.S., Liu X., Chua A.N., Wang W., McIntyre R.S., Ho R.C. (2017). Chronic administration of fluoxetine and pro-inflammatory cytokine change in a rat model of depression. PLoS ONE.

[147] Cui C., Shurtleff D., Harris R.A. (2014). Neuroimmune mechanisms of alcohol and drug addiction. Int. Rev. Neurobiol..

[148] Hodes G.E., Kana V., Menard C., Merad M., Russo S.J. (2015). Neuroimmune mechanisms of depression. Nat. Neurosci..

[149] Tzingounis A.V., Wadiche J.I. (2007). Glutamate transporters: Confining runaway excitation by shaping synaptic transmission. Nat. Rev. Neurosci..

[150] Wohleb E.S., Franklin T., Iwata M., Duman R.S. (2016). Integrating neuroimmune systems in the neurobiology of depression. Nat. Rev. Neurosci..

[151] Versace V., Sebastianelli L., Ferrazzoli D., Romanello R., Ortelli P., Saltuari L., D’Acunto A., Porrazzini F., Ajello V., Oliviero A. (2021). Intracortical GABAergic dysfunction in patients with fatigue and dysexecutive syndrome after COVID-19. Clin. Neurophysiol..

[152] Furlanis G., Buoite Stella A., Biaduzzini F., Bellavita G., Frezza N.A., Olivo S., Menichelli A., Lunardelli A., Ajčević M., Manganotti P. (2023). Cognitive deficit in post-acute COVID-19: An opportunity for EEG evaluation?. Neurol. Sci..

[153] Yesilkaya U.H., Sen M., Balcioglu Y.H. (2021). COVID-19-related cognitive dysfunction may be associated with transient disruption in the DLPFC glutamatergic pathway. J. Clin. Neurosci..

[154] Rapalino O., Weerasekera A., Moum S.J., Eikermann-Haerter K., Edlow B.L., Fischer D., Torrado-Carvajal A., Loggia M.L., Mukerji S.S., Schaefer P.W. (2021). Brain MR Spectroscopic Findings in 3 Consecutive Patients with COVID-19: Preliminary Observations. AJNR Am. J. Neuroradiol..

[155] Newhouse A., Kritzer M.D., Eryilmaz H., Praschan N., Camprodon J.A., Fricchione G., Chemali Z. (2022). Neurocircuitry hypothesis and clinical experience in treating neuropsychiatric symptoms of postacute sequelae of severe acute respiratory syndrome coronavirus 2. J. Acad. Consult. Liaison Psychiatry.

[156] Baig A.M. (2022). Differential diagnosis and pathogenesis of the neurological signs and symptoms in COVID-19 and long-COVID syndrome. CNS Neurosci. Ther..

[157] Bookstaver P.B., Mohorn P.L., Shah A., Tesh L.D., Quidley A.M., Kothari R., Bland C.M., Weissman S. (2017). Management of viral central nervous system infections: A primer for clinicians. J. Cent. Nerv. Syst. Dis..

[158] Schifitto G., Navia B.A., Yiannoutsos C.T., Marra C.M., Chang L., Ernst T., Jarvik J.G., Miller E.N., Singer E.J., Ellis R.J. (2007). Memantine and HIV-associated cognitive impairment: A neuropsychological and proton magnetic resonance spectroscopy study. Aids.

[159] Gonul A.S., Kitis O., Ozan E., Akdeniz F., Eker C., Eker O.D., Vahip S. (2006). The effect of antidepressant treatment on N-acetyl aspartate levels of medial frontal cortex in drug-free depressed patients. Prog. Neuro-Psychopharmacol. Biol. Psychiatry.

[160] Huang Y., Chen W., Li Y., Wu X., Shi X., Geng D. (2010). Effects of antidepressant treatment on N-acetyl aspartate and choline levels in the hippocampus and thalami of post-stroke depression patients: A study using 1H magnetic resonance spectroscopy. Psychiatry Res. Neuroimaging.

[161] Patel T., Blyth J.C., Griffiths G., Kelly D., Talcott J.B. (2014). Moderate relationships between NAA and cognitive ability in healthy adults: Implications for cognitive spectroscopy. Front. Hum. Neurosci..

